# Oligodendroglioma Cells Lack Glutamine Synthetase and Are Auxotrophic for Glutamine, but Do not Depend on Glutamine Anaplerosis for Growth

**DOI:** 10.3390/ijms19041099

**Published:** 2018-04-06

**Authors:** Martina Chiu, Giuseppe Taurino, Massimiliano G. Bianchi, Laura Ottaviani, Roberta Andreoli, Tecla Ciociola, Costanza A. M. Lagrasta, Saverio Tardito, Ovidio Bussolati

**Affiliations:** 1 Laboratory of General Pathology, Department of Medicine and Surgery, University of Parma, Via Volturno 39, 43125 Parma, Italy; martina.chiu@unipr.it (M.C.); giuseppe.taurino@studenti.unipr.it (G.T.); massimiliano.bianchi@unipr.it (M.G.B.); laura.ottaviani986@gmail.com (L.O.); 2 Laboratory of Industrial Toxicology, Department of Medicine and Surgery, University of Parma, Via Gramsci 14, 43126 Parma, Italy; roberta.andreoli@unipr.it; 3 Laboratory of Medical Microbiology and Virology, Department of Medicine and Surgery, University of Parma, Via Volturno 39, 43125 Parma, Italy; tecla.ciociola@unipr.it; 4 Laboratory of Anatomical Pathology, Department of Medicine and Surgery, University of Parma, Via Gramsci 14, 43126 Parma, Italy; costanzaannamaria.lagrasta@unipr.it; 5 Cancer Research UK Beatson Institute, Garscube Estate, Switchback road, Glasgow G611BD, UK; s.tardito@beatson.gla.ac.uk

**Keywords:** anaplerosis, beta-catenin, glutamine, glutamine addiction, glutamine transport, oligodendroglioma

## Abstract

In cells derived from several types of cancer, a transcriptional program drives high consumption of glutamine (Gln), which is used for anaplerosis, leading to a metabolic addiction for the amino acid. Low or absent expression of Glutamine Synthetase (GS), the only enzyme that catalyzes de novo Gln synthesis, has been considered a marker of Gln-addicted cancers. In this study, two human cell lines derived from brain tumors with oligodendroglioma features, HOG and Hs683, have been shown to be GS-negative. Viability of both lines depends from extracellular Gln with EC_50_ of 0.175 ± 0.056 mM (Hs683) and 0.086 ± 0.043 mM (HOG), thus suggesting that small amounts of extracellular Gln are sufficient for OD cell growth. Gln starvation does not significantly affect the cell content of anaplerotic substrates, which, consistently, are not able to rescue cell growth, but causes hindrance of the Wnt/β-catenin pathway and protein synthesis attenuation, which is mitigated by transient GS expression. Gln transport inhibitors cause partial depletion of intracellular Gln and cell growth inhibition, but do not lower cell viability. Therefore, GS-negative human oligodendroglioma cells are Gln-auxotrophic but do not use the amino acid for anaplerosis and, hence, are not Gln addicted, exhibiting only limited Gln requirements for survival and growth.

## 1. Introduction

Glutamine (Gln), the most abundant amino acid in blood, fuels several metabolic processes that sustain the growth of normal and cancer cells. In particular, in cells derived from several types of cancer a transcriptional program drives high consumption of Gln, which is readily hydrolyzed to glutamate (Glu) by glutaminases (GLS and GLS-2) [1,2,3]. In these Gln-addicted cancer cells, Glu is further deaminated or transaminated to 2-oxoglutarate (2-OG), an intermediate of the tricarboxylic acid cycle (TCA) [1,2]. Thus, Gln can contribute five carbons to the TCA cycle, thereby constituting an optimal anaplerotic substrate. Moreover, Gln-derived Glu contributes to the biosynthesis of glutathione (GSH), sustaining cell redox equilibrium [4]. Besides anaplerosis and GSH synthesis, Gln is essential for the biosynthesis of asparagine, nucleotides, and glucosamine 6-phosphate. Finally, Gln promotes mTORC1 activity, thus inhibiting autophagy [5,6] and, more recently, has been found to affect gene expression independently of its metabolism [7].

Gln-addicted cancer cells, when incubated in glutamine-free medium, or treated with the glutamine-depleting enzyme L-asparaginase, undergo a severe nutritional stress that can lead to cell death [8,9,10,11]. These cells usually express high levels of one or more Gln transporters. Indeed, several transporters, such as the sodium-dependent SNAT1-2, ASCT2, and ATB0^+^, as well as the sodium-independent LAT1, can mediate glutamine uptake by cancer cells [12]. For instance, SNAT1 and 2 are known to sustain glutaminolysis in HeLa cells [13]. Other cancer cells overexpress LAT1 and ASCT2 [13,14], the inhibition of which severely hinders the growth of glutamine-dependent tumors [9,15,16,17].

Glutamine Synthetase (GS), encoded by *GLUL*, is the only enzyme able to catalyze biosynthesis of Gln from Glu and ammonia. Through GS activity, cells can counteract the effects of Gln depletion, although the rescuing effect of GS varies considerably among different cancer cell models, and GS overexpression is insufficient to sustain cell growth under Gln deprivation in some cancer cells [10,18]. Interestingly, even in the absence of Gln depletion, the sole inhibition of GS is able to hinder the growth of human hepatocellular carcinoma murine xenografts [11].

Oligodendroglioma (OD) is a brain tumor of glial origin. In the large majority of patients, OD present with 1p/19q co-deletions [19] and mutations in the *IDH1* gene [20]. Moreover, for the most part, ODs are histologically negative for GS [21,22]. While stable cultures of OD cells mutant for *IDH1* have not been obtained thus far, no information is available on GS expression in cultured OD cells, although the original report on the isolation of the HOG cell line reported that these cells had no significant GS activity [23]. However, although both IDH1 and GS are relevant to Gln metabolism, the effects of Gln restriction have not been yet investigated in human OD cells.

Here we show that two cell lines derived from human ODs lack a sizable GS expression, do not exhibit Gln-dependent anaplerosis, reduce proliferation upon Gln restriction, and undergo apoptosis upon complete Gln deprivation.

## 2. Results

### 2.1. Oligodendroglioma Cells Lack Glutamine Synthetase and Die Upon Glutamine Withdrawal

**Figure 1:**
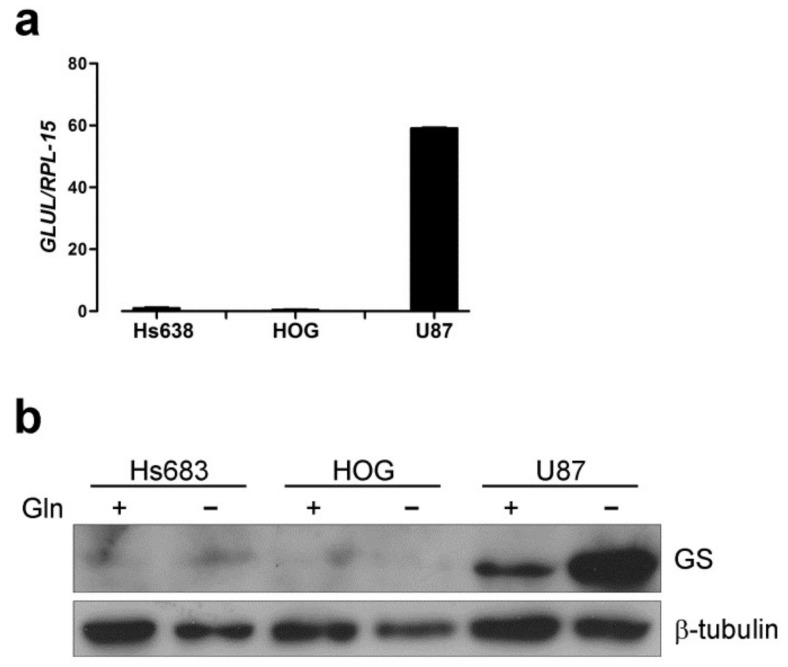
Glutamine Synthetase expression is negligible in human oligodendroglioma cells. (**a**) *GLUL* mRNA expression was assessed by real-time PCR in Hs683, HOG and U87 cells incubated in standard growth medium ([Gln] = 4 mM). Data were normalized to the expression of *RPL-15*. (**b**) Western blot of GS in Hs683, HOG and U87 cells. Cells were incubated in the presence (+) or in the absence (−) of Gln for 19 h. β-tubulin was used for loading control. A representative experiment is shown.

Firstly, we verified whether the human oligodendroglioma (OD) cell lines Hs683 and HOG express Glutamine Synthetase (GS). When compared with the human glioblastoma cell line U87, OD cells expressed much less GS at mRNA and protein levels (Figure 1a,b). In line with previous reports [24,25], incubation in a Gln-free medium increased the expression of GS protein in U87 but not in OD cells (Figure 1b).

**Figure 2:**
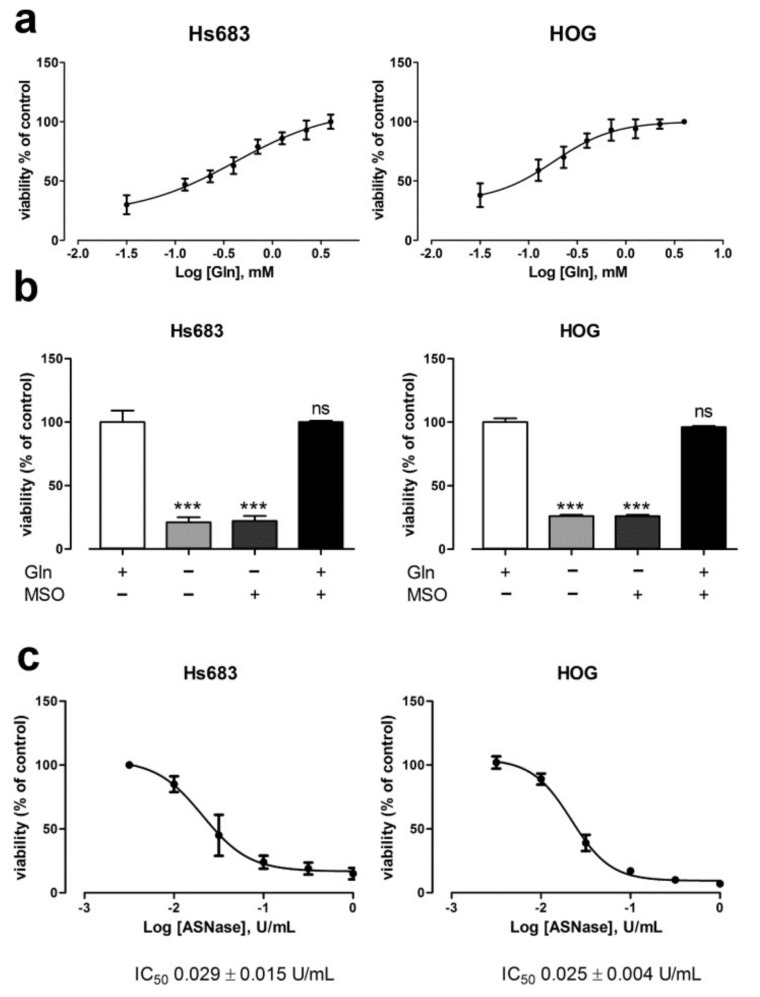

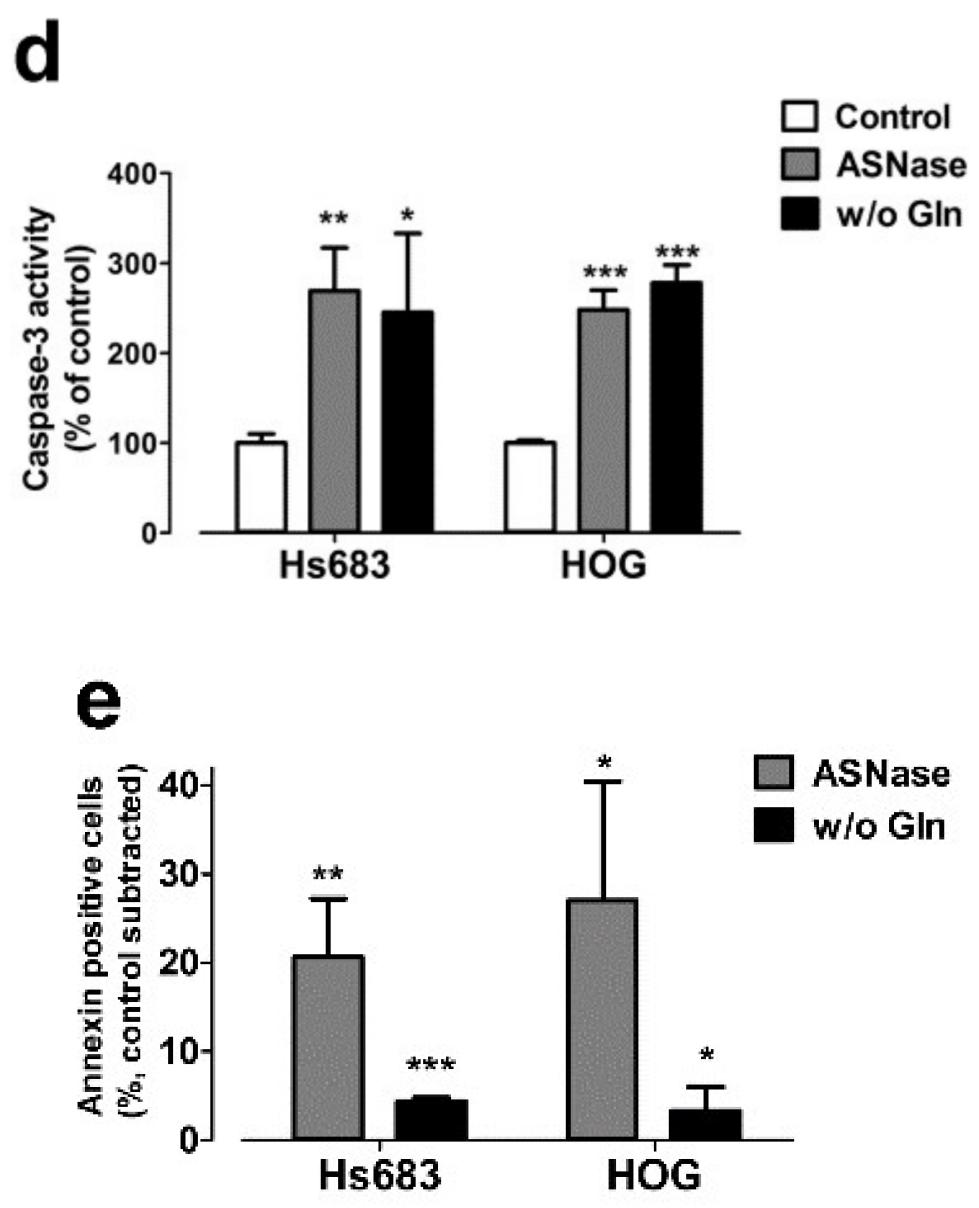
Gln starvation kills oligodendroglioma cells. (**a**) Viability of Hs683 and HOG cells incubated with increasing concentrations of Gln (0.03, 0.125, 0.25, 0.4, 0.7, 1.25, 2 and 4 mM) for 48 h. Data are expressed as % of control (4 mM Gln). Means ± SD of three experiments, with three independent determinations each, are shown. Dose response curves were evaluated by non-linear regression analysis. (**b**) Viability of Hs683 and HOG cells incubated in the presence (+) or in the absence (−) of Gln (4 mM), or MSO (1 mM) for 48 h. Data are expressed as % of control (cells maintained at 4 mM Gln). Means ± SD of three experiments, with three independent determinations each, are shown. *** *p* < 0.001 vs. control, ns, not significant, as assessed with a two-tail Student *t* test for unpaired data. (**c**) Viability of Hs683 and HOG cells incubated with increasing concentrations of L-asparaginase (0.003, 0.01, 0.03, 0.1, 0.3, and 1 U/mL) for 48 h. Data are expressed as % of control (untreated cells). Means ± SD of three experiments, with three independent determinations each, are shown. Dose response curves were evaluated by non-linear regression analysis. (**d**) Caspase-3 activity was assessed in Hs683 and HOG cells treated for 36 h in the presence (Control) or in the absence (*w*/*o* Gln) of Gln (4 mM) or in the presence of L-asparaginase (ASNase, 1 U/mL). (**e**) Annexin V positive population was evaluated in Hs683 and HOG cells treated for 24 h as described in panel c. The graph shows the mean % plus SD (*n* = 3) of Annexin V positive cells for each condition after the subtraction of the value obtained in control. For (**c**,**d**), data represent means ± SD of two experiments with two independent determinations each.* *p* < 0.05, ** *p* < 0.01, *** *p* < 0.001, as assessed with a two-tail Student *t* test for unpaired data.

Given that OD cells express very low levels of GS, we hypothesized that they require large amounts of extracellular Gln for growth. To test this hypothesis, firstly we incubated OD cells with decreasing concentration of Gln. Unexpectedly, both OD cells showed low values of EC_50_ for Gln (Figure 2a, 0.175 ± 0.056 mM (Hs683) and 0.086 ± 0.043 mM (HOG)), thus suggesting that relatively small amounts of extracellular Gln are sufficient for OD cell growth. However, in the absence of Gln, cell viability of both Hs683 and HOG cells was severely affected (Figure 2b). In line with the extremely low expression of GS, MSO, an irreversible inhibitor of GS, did not further reduce the viability of Gln-starved OD cells, while it was without any effect in Gln-fed cells (Figure 2b). Moreover, L-asparaginase, an enzyme that converts the extracellular Gln into glutamate (Glu), decreased the viability of OD cells in a dose dependent manner (Figure 2c, IC_50_ = 0.029 ± 0.015 U/mL (Hs683) and 0.025 ± 0.004 U/mL (HOG)).

l-Asparaginase, as well as the incubation in Gln-free medium, increased the levels of caspase-3 activity and the percentage of annexin V positive cells, two markers of apoptosis (Figure 2d,e).

### 2.2. The Depletion of Anaplerotic Substrates and GSH Does not Explain the Effect of Gln Starvation on the Viability of OD Cells

**Figure 3:**
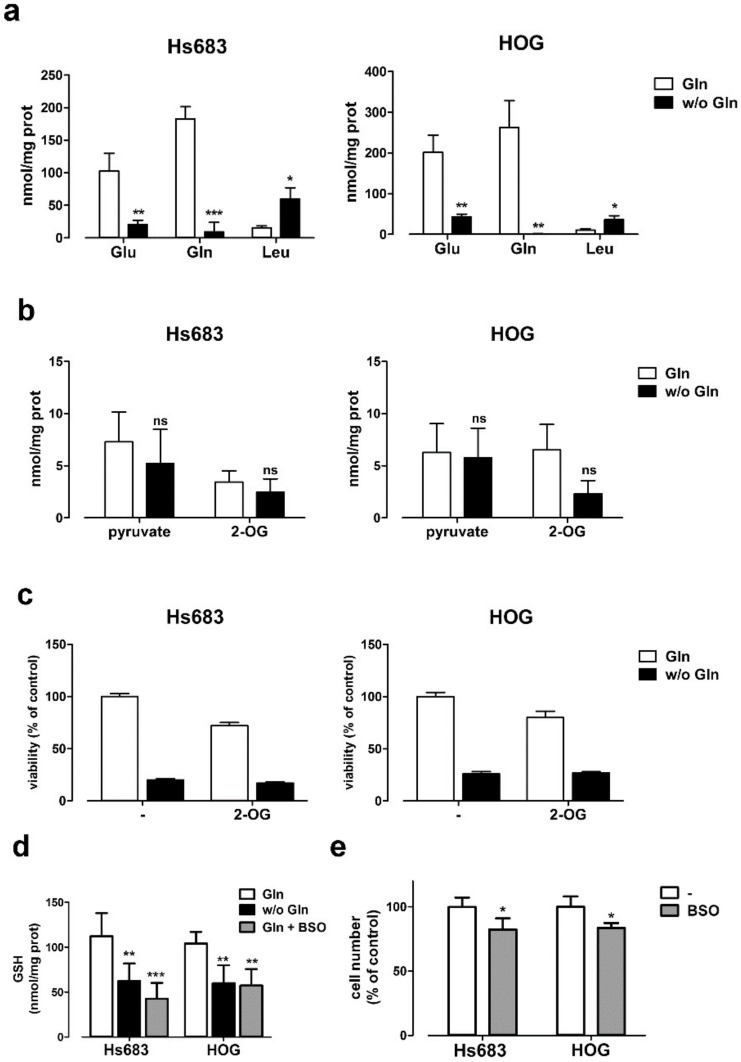
Oligodendroglioma cell viability does not correlate with changes in anaplerotic substrates or GSH. (**a**,**b**) Cell contents of Glu, Gln, and Leu (**a**) or of pyruvate and 2-OG (**b**) were measured in Hs683 and HOG cells incubated for 19 h in the presence (Gln, 4 mM) or in the absence (*w*/*o* Gln) of Gln. Data are expressed as nmol/mg protein. Means ± SD of three experiments are shown. * *p* < 0.05, ** *p* < 0.01, *** *p* < 0.001, ns, not significant, as assessed with one-way ANOVA. (**c**) Viability of Hs683 and HOG cells incubated for 48 h in the presence (Gln) or in the absence (*w*/*o* Gln) of Gln (4 mM), with or without a membrane-permeant form of 2-OG (dimethyl-2-oxoglutarate, 8 mM). Means ± SD of three experiments, with three independent determinations each, are shown. (**d**) The cell content of reduced GSH in Hs683 and HOG cells incubated for 19 h in the presence (4 mM) or in the absence of Gln (*w*/*o* Gln). Data are expressed as nmol/mg protein. Means ± SD of three experiments are shown. ** *p* < 0.01, *** *p* < 0.001, ns, not significant, as assessed with one-way ANOVA. (**e**) Viability of Hs683 and HOG cells treated for 48 h with BSO (1 mM). Means ± SD of three experiments, with three independent determinations each, are shown. * *p* < 0.05, as assessed with student *t* test for unpaired data.

In several human cancer models, Gln sustains cell growth through anaplerosis [1,4,26]. To test whether also OD cells depend on Gln contribution to replenish the TCA cycle, the intracellular content of pyruvate and 2-oxoglutarate (2-OG), two anaplerotic substrates, was measured by LC-MS/MS in either Gln-fed or Gln-starved cells. First, in the absence of Gln, the intracellular levels of Gln and Glu were markedly decreased in both OD cell lines, while the intracellular levels of leucine were increased (Figure 3a). Under the same conditions, pyruvate and 2-oxoglutarate were not significantly reduced by Gln removal (Figure 3b), although a trend towards a decrease in 2-oxoglutarate was shown especially by HOG cells. However, the supplementation with dimethyl-oxoglutarate, a membrane-permeable form of 2-OG, did not rescue the viability of Gln-starved cells (Figure 3c).

Another important metabolic endpoint for glutamine is the incorporation of Gln-derived glutamate into glutathione (GSH). Indeed, Gln starvation caused a significant decrease in the intracellular content of GSH in OD cells, which was comparable to that obtained with BSO, an inhibitor of GSH synthesis. However, BSO produced only a modest effect on OD cell number (~15%), much smaller than that caused by Gln withdrawal (~80%), suggesting that the reduced levels of glutathione only marginally account for the drop in viability observed upon Gln starvation. Altogether, these results indicate that Gln sustains growth and survival of OD cells through a mechanism that is largely independent from the synthesis of TCA cycle intermediates and glutathione.

### 2.3. Gln-Starvation Induces a Nutritional Stress Response and Hinders mTOR and Wnt/β-Catenin Pathways in OD Cells

**Figure 4:**
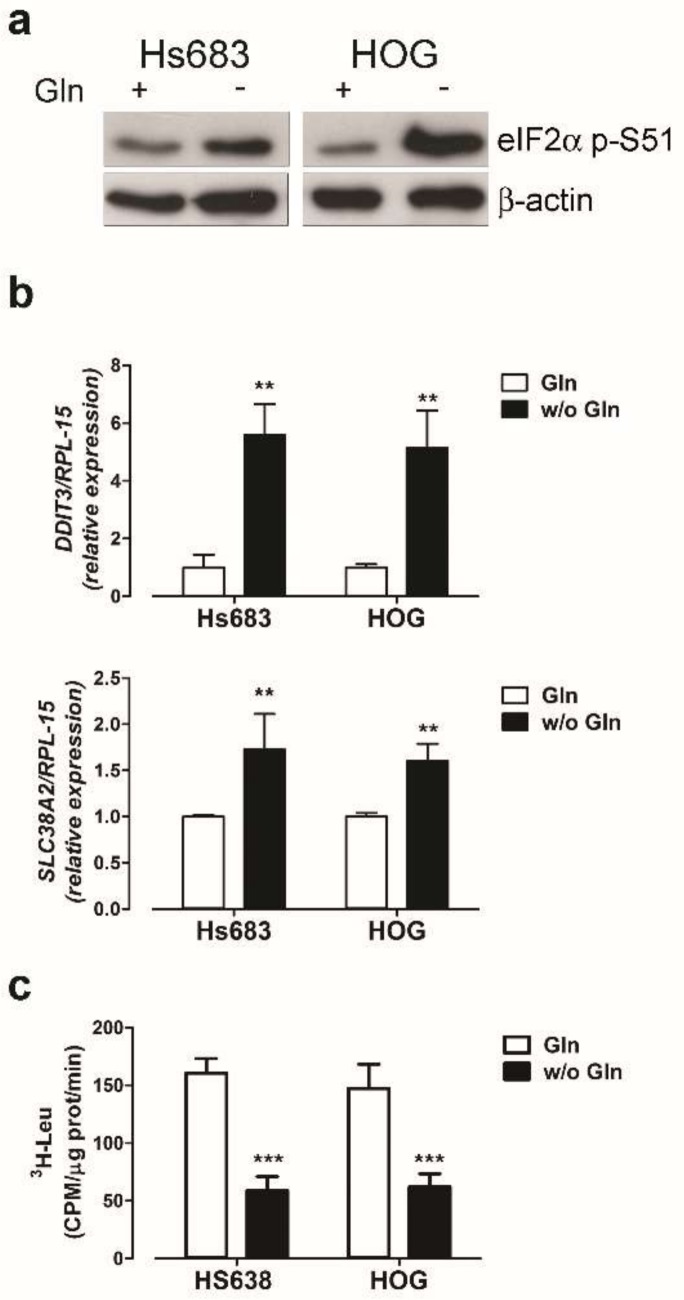

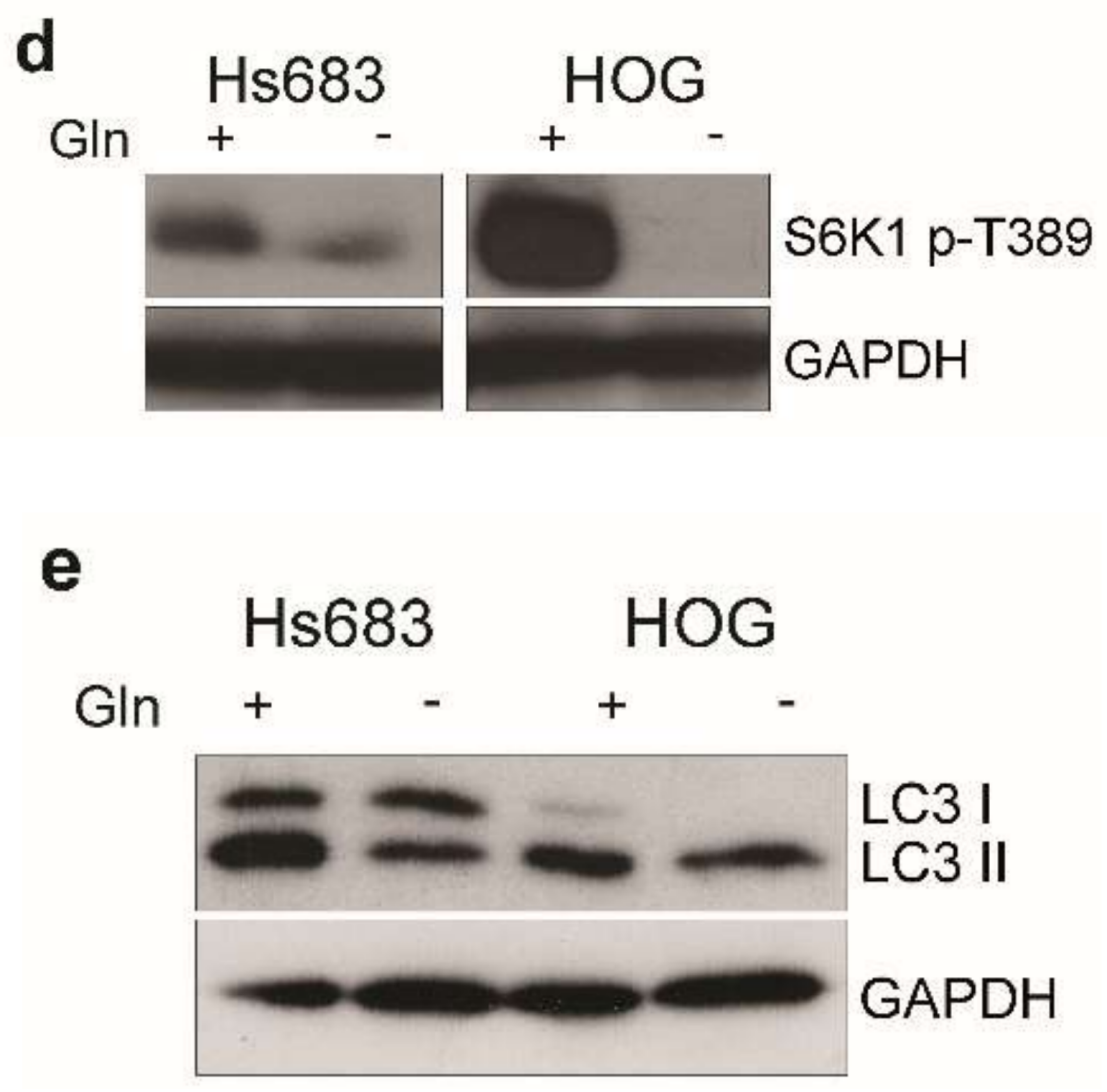
Gln starvation triggers a nutritional stress response. (**a**–**e**) Hs683 and HOG cells were incubated in the presence (+) or in the absence (−) of Gln. (**a**) Western blot of eIF2α (p-S51) upon a 1h-incubation. β-actin was used for loading control. (**b**) *DDIT3* and *SLC38A2* mRNA expression was assessed by real-time PCR upon a 19h-Gln-free-incubation. Data were normalized to the expression of *RPL-15*. (**c**) At the same time the rate of protein synthesis was determined as described in Methods. Data are expressed as CPM/μg prot/min. (**d**) Western blot of p70S6K1 (T389) after an incubation of 6 h. GAPDH was used for loading control. (**e**) Western blot of LC3 after an incubation of 19 h in the presence or in the absence of Gln. GAPDH was used for loading control. For (**a**,**d**,**e**) representative experiments are shown. For (**b**,**c**) means ± SD of three experiments are shown. ** *p* < 0.01, *** *p* < 0.001, as assessed with two-tail Student *t* test for unpaired data.

In both OD cell lines, the incubation in a Gln-free medium caused an increase in the phosphorylation of the α subunit of the eukaryotic initiator factor 2 (eIF2α), a marker of nutritional stress (Figure 4a). In response to the nutritional stress imposed by Gln starvation, the genes encoding for the pro-apoptotic protein CHOP (*DDIT3*) and the amino acid transporter SNAT2 (*SLC38A2*) were markedly induced (Figure 4b), while protein synthesis rate was reduced (Figure 4c). Moreover, Gln depletion hindered mTORC1 activity (Figure 4d), as assessed from the phosphorylation of its downstream target S6K1 (Figure 4d). Unexpectedly, the inhibition of mTORC1 was not associated with an increase of the autophagic marker LC3 II (Figure 4e), which was in fact also well evident in Gln-fed cells.

**Figure 5:**
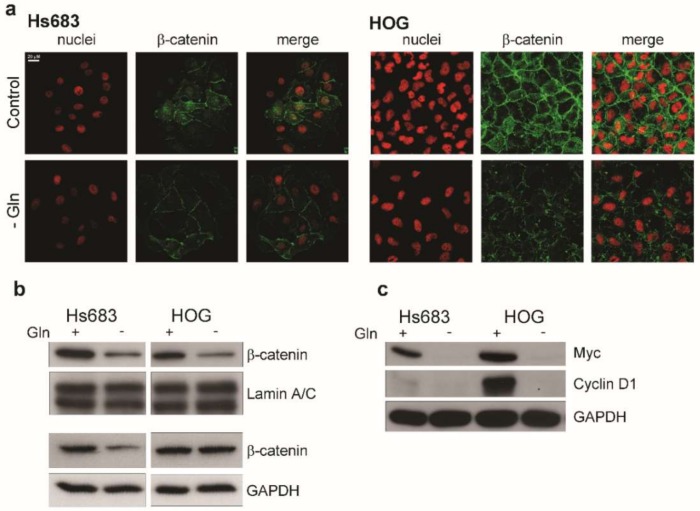
Gln starvation inhibits the Wnt/β-catenin pathway. (**a**–**c**) Hs683 and HOG cells incubated in the presence (Control, +) or in the absence (- Gln, −) of Gln for 19 h. (**a**) Immunofluorescence of β-catenin. Single confocal sections of representative fields. Red, nuclei, Propidium iodide; Green, β-catenin. Bar = 20 μm (**b**) Western blot of β-catenin in nuclear (above) or in cytosolic (below) extracts of Hs683 and HOG cells. Lamin A/C or GAPDH were used for loading control. (**c**) Western blot of Myc and cyclin D1. GAPDH was used for loading control. Representative experiments are shown.

Since OD cell proliferation has been found to depend on an active Wnt/β-catenin signaling pathway [27], we next looked at β-catenin expression and localization. Upon Gln starvation, a reduction in the β-catenin expression was observed in confocal microscopy (Figure 5a). Cytoplasmic fraction of β-catenin decreased only in Hs683 cells, while a marked decrease of the abundance of the nuclear, transcriptionally active fraction was observed in both cell lines (Figure 5b). Consistently, the expression of two β-catenin targets, c-myc and cyclin D1, was markedly reduced by Gln deprivation either in Hs683 or in HOG cells (Figure 5c).

### 2.4. Glutamine Synthetase Expression Mitigates the Nutritional Stress Induced by Gln Starvation

**Figure 6:**
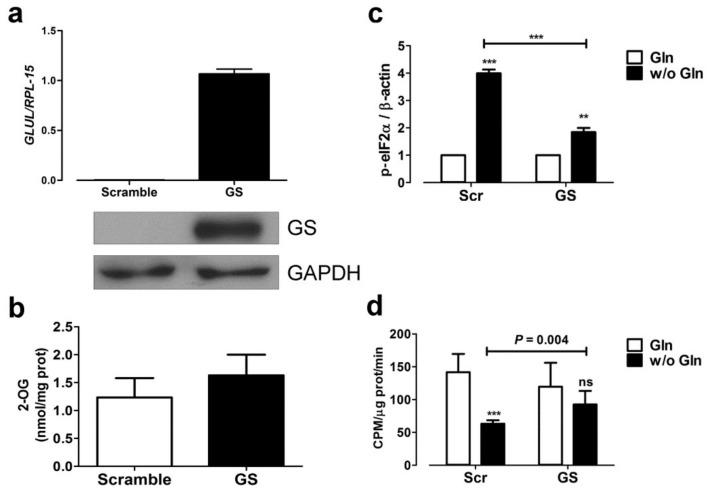
GS expression blunted eIF2α phosphorylation and rescued protein synthesis in Gln-starved cells. (**a**,**b**) *GLUL* mRNA (**a**, **top**), GS protein (**a**, **bottom**), and 2-OG content (**b**) were assessed by real-time PCR (**a**, **top**), Western Blot (**a**, **bottom**) or LC-MS/MS (**b**) in Hs683 cells transfected with pCMV-GLUL vector (GS) or a scramble control (Scramble). PCR data were normalized to the expression of *RPL-15*, and GAPDH was used for loading control for Western Blot. A representative experiment is shown. (**c**,**d**) Hs683 cells, transfected with the pCMV-GLUL vector (GS) or a scramble control (Scr), were incubated with or without Gln. (**c**) After 1 h, the expression of the phosphorylated form of eIF2α was assessed. β-actin was used for loading control. The graph shows the relative expression (means ± SD) of p-eIF2α obtained in three independent experiments. ** *p* < 0.01, *** *p* < 0.001, as assessed with two-tail student *t* test. (**d**) After 19 h, the rate of protein synthesis 0was assessed. Data are expressed as CPM/μg prot/min. Means ± SD of three experiments are shown. *** *p* < 0.001, as assessed with two-way ANOVA; ns, not significant.

Next, we forced the expression of GS in Hs683 cells by transiently transfecting a vector where GS expression is driven by a CMV promoter (Figure 6a). GS overexpression did not significantly modify 2-OG content (Figure 6b), but lowered the levels of the phosphorylated form of eIF2α in Gln depleted cells upon Gln starvation (Figure 6c). Consistently, in GS expressing cells the protein synthesis was not significantly different with and without Gln, while in control cells the rate of protein synthesis was halved by Gln removal (Figure 6d).

### 2.5. Oligodendroglioma Cells Transport Glutamine through Several Systems

**Figure 7:**
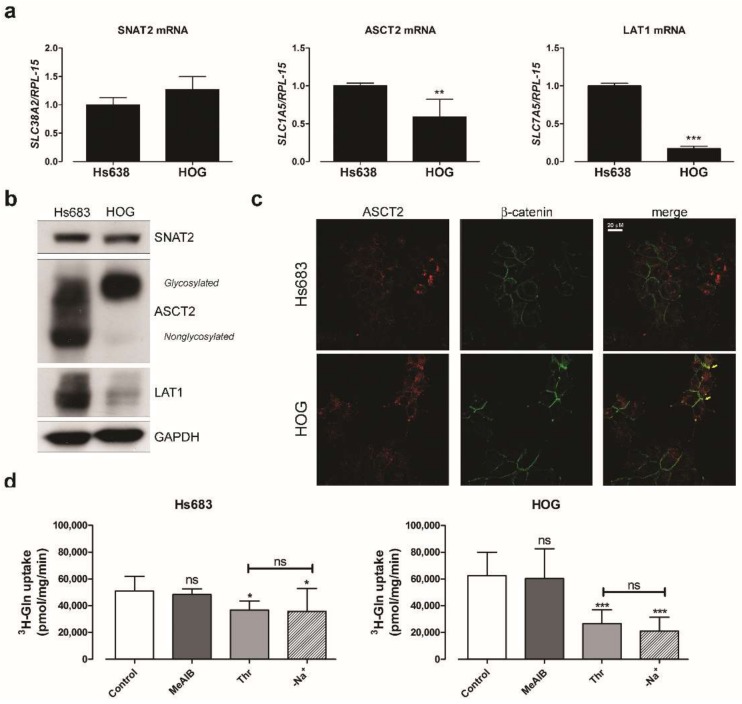

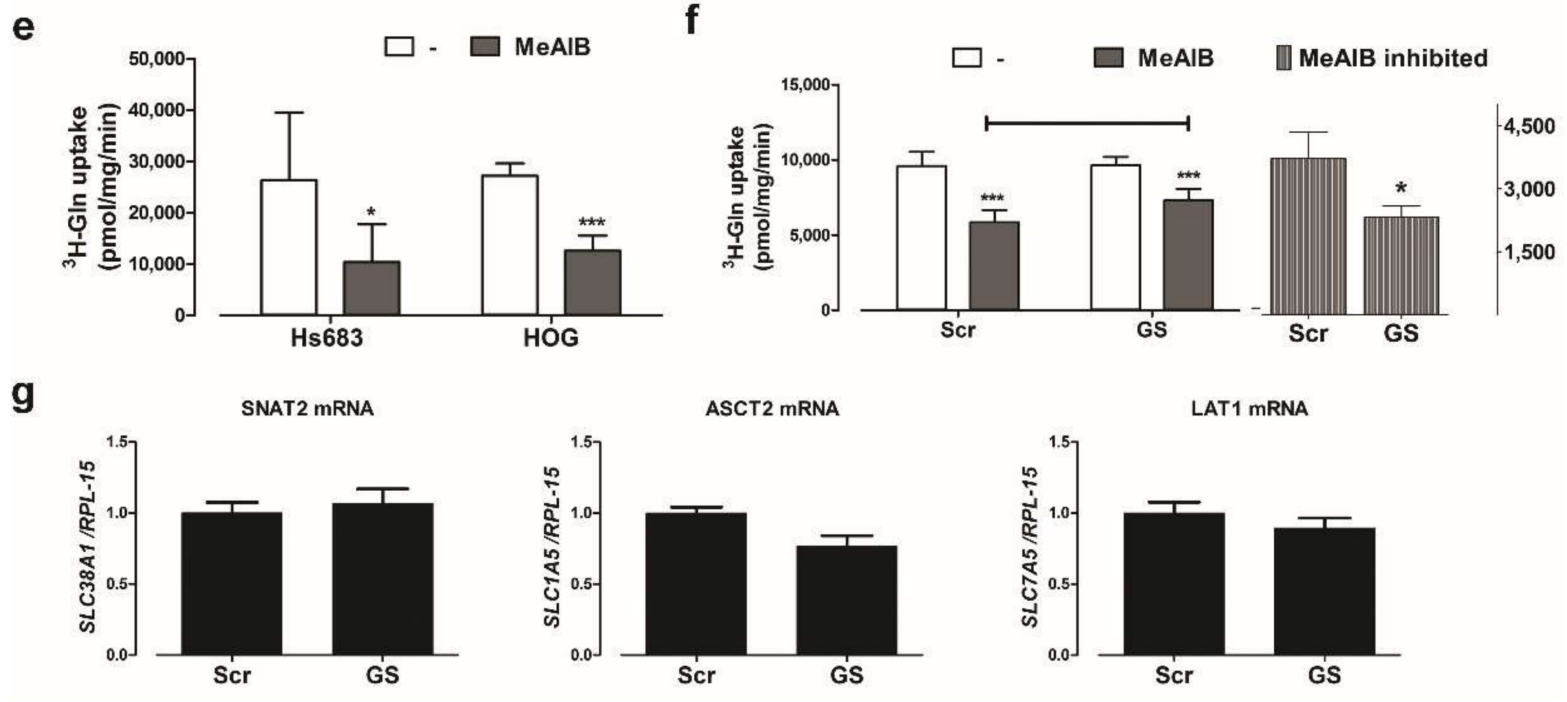
Human OD cells transport Gln through several carriers (**a**) *SLC38A2, SLC1A5* and *SLC7A5* mRNA expression was assessed in Hs683 and HOG cells. Data were normalized to the expression of *RPL-15*. (**b**) Western blot of SNAT2, ASCT2 and LAT1 in Hs683 and HOG cells. GADPH was used as loading control. (**c**) Immunofluorescence of ASCT2 (red) in Hs683 and HOG cells. β-catenin (green) was used as a marker of cell periphery. (**d**) 1-Min uptake of Gln (0.6 mM, 5 μCi/mL) performed in EBSS in the absence (Control) or in the presence of MeAIB (20 mM), threonine (Thr, 5 mM), or in Na^+^-free EBSS (-Na^+^). (**e**) 1-Min uptake of Gln (2 mM, 5 μCi/mL) performed in Hs683 and HOG cells pre-incubated in Gln-free EBSS for 30 min. Gln uptake was performed in the absence or in the presence of MeAIB (20 mM). (**f**) 1-Min uptake of Gln (2 mM, 5 μCi/mL) was performed in Hs683 cells transfected with pCMV-*GLUL* vector (GS) or a scramble control (Scr) incubated after a 30 min incubation in Gln-free EBSS. Gln uptake was performed in the absence or in the presence of MeAIB (20 mM). (*right*) MeAIB-inhibited fraction was calculated by subtracting the uptake in the presence of MeAIB to the corresponding total Gln uptake. (**g**) *SLC38A2, SLC1A5* and *SLC7A5* mRNA expression was assessed in scramble (Scr)- and *GLUL*-transfected (GS) Hs683 cells. Data were normalized to the expression of *RPL-15*. For (**a**,**g**), means ± SD of three experiments are shown. ** *p* < 0.01, *** *p* < 0.001, as assessed with two-tail student *t* test. For (**d**–**f**), data are expressed as pmol/mg prot/min and are means ± SD of three (for (**d**,**e**)) or two (for (**f**)) experiments, with five independent determinations each. For (**d**,**e**), ** *p* < 0.01, *** *p* < 0.001, ns, not significant, as assessed with two-tail student *t* test; for (**f**), ** *p* < 0.01, as assessed with two-way ANOVA.

We demonstrated that OD cells rely on extracellular Gln for survival; therefore, we investigated which transport systems are responsible for Gln influx in these cells. We assessed the expression of *SCL38A2*, *SLC7A5*, *SCL1A5*, which encode, respectively, the transporters SNAT2 (System A), LAT1 (System L), ASCT2 (System ASC). All these carriers were expressed in both cell lines (Figure 7a), with Hs683 cells expressing significantly more *SLC1A5* and *SLC7A5* than HOG cells at mRNA and protein levels (Figure 7b). However, while ASCT2 band was detected at the molecular weight expected for the glycosylated form in HOG cells [28], Hs683 cells mainly expressed non-glycosylated forms of ASCT2 (Figure 7b). Consistently with this glycosylation pattern, peripheral co-localization of ASCT2 with β-catenin was detectable in HOG cells, but not in Hs683 cells (Figure 7c).

To evaluate the relationship between expression and function of the transport systems we performed an uptake assay using ^3^H-Gln. At 0.6 mM, a concentration comparable with that present in human plasma, Gln was transported at similar rates in the two cell lines (Figure 7d). Consistently with the high LAT1 expression detected in Hs683 cells, the Na^+^-independent influx of Gln was higher in Hs683 than in HOG cells (Figure 7d). The activity of ASCT2, evaluated from the threonine-inhibitable fraction of Gln uptake, was instead higher in HOG cells than in Hs683 cells. However, in both cell lines, the Na^+^-dependent uptake of Gln was completely suppressed by threonine, the preferential substrate of ASCT2 [29], indicating that this transporter is largely responsible for the Na^+^-dependent uptake of Gln in OD cells.

MeAIB is a specific inhibitor of the system A transporter SNAT2. At 0.6 mM Gln, the inhibitor did not reduce Gln uptake in cells maintained in culture medium until the transport assay. However, if the assay was performed after incubating cells for 30 min in a Gln-free saline solution, MeAIB significantly inhibited Gln transport in both cell lines. This result is expected, given the membrane translocation of SNAT2 upon amino acid depletion [30], and suggests that this transporter contributes to Gln uptake in starved cells (Figure 7e).

Interestingly, upon Gln withdrawal, the portion of Gln uptake attributable to SNAT2 (MeAIB-inhibited fraction) was larger in scramble-transfected than in GS-expressing Hs683 cells (Figure 7f), suggesting that the nutritional stress is less severe in the latter condition. Under basal conditions (Gln present), the expression of SNAT2 mRNA, like that of ASCT2 and LAT1, was not modified by *GLUL* transfection (Figure 7g).

**Figure 8:**
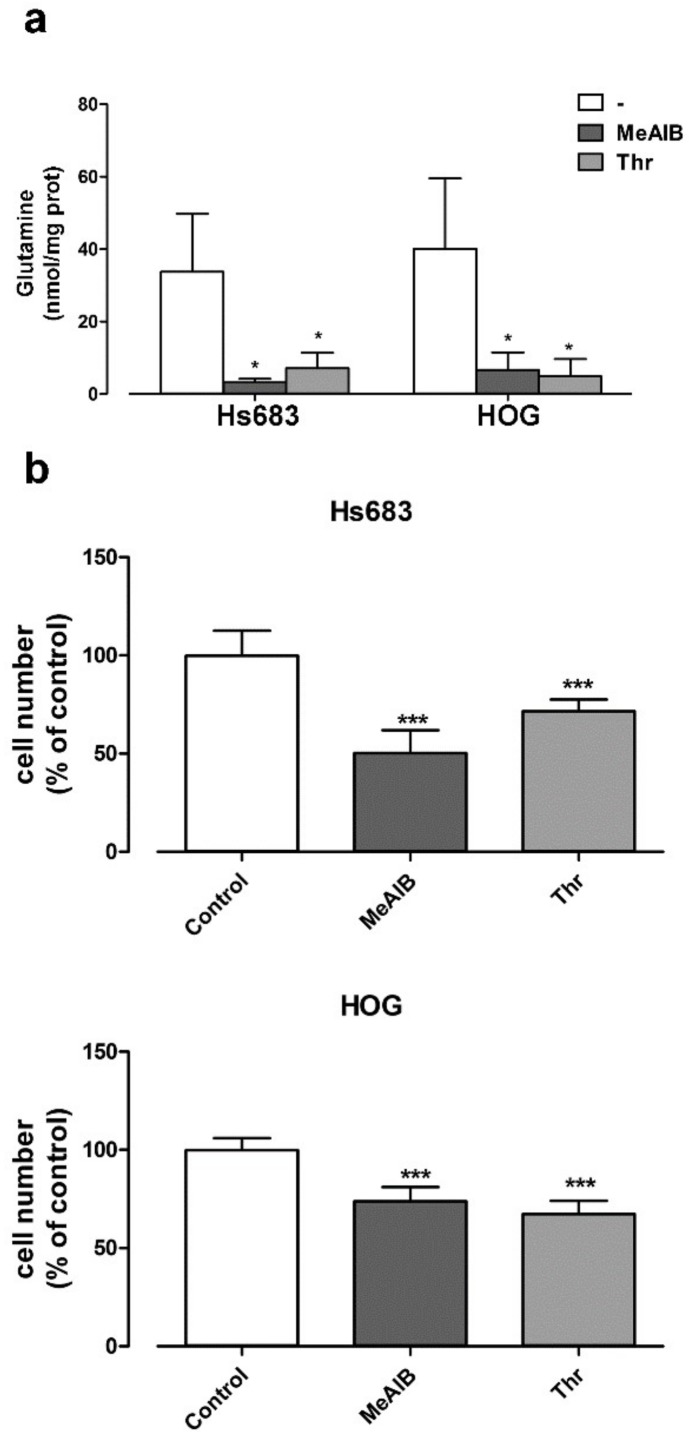
Inhibitors of Gln transporters have anti-proliferative effects on oligodendroglioma cells. Hs683 and HOG cells were incubated in growth medium in the absence (Control) or in the presence of MeAIB (20 mM) or Thr (20 mM). (**a**) After 9 h, the cell content of Gln was measured. Data are expressed as nmol/mg prot. (**b**) After 72 h, the cell number was evaluated. Data are expressed as % of control (standard growth medium). In both panels, data are means ± SD of three experiments with three independent determinations each. * *p* < 0.05, *** *p* < 0.001, as assessed with two-tail student *t* test for unpaired data.

Lastly, we investigated the effects of Gln-transport inhibitors on OD cells. A 9h-incubation in the presence of 20 mM Thr (the preferential ASCT2 substrate), or 20 mM MeAIB (the SNAT2 inhibitor) decreased the cell content of Gln by more than 80%. The substantial reduction in the intracellular Gln pool was associated with a 25% decrease in cell proliferation compared to untreated cells (Figure 8b) but did not cause apoptosis (not shown). This suggests that the inhibition of Gln transport, while limiting cell growth, is not sufficient to trigger cell death.

## 3. Discussion

In this contribution we have studied the effects of Gln depletion on two human oligodendroglioma (OD) cell lines, HOG and Hs683. Although these cells present neither *IDH1* mutation nor 1p19q co-deletion, two molecular markers of ODs in vivo, this does not contradict their OD origin, since it is controversial whether a stable glioma cell line with these features can be established, and, moreover, the radio-chemosensitivity of these cell models is similar to that of primary ODs [20,31,32,33]. However, both lines have very low levels of expression of Glutamine Synthetase (GS), another characteristic commonly found in human ODs [21,22]. While amino acid metabolism has been investigated in either Hs683 or HOG cells [31,34], the effects of Gln deprivation have never been studied in these cells models.

Gln-addicted cancers are extremely sensitive to Gln depletion, utilize large quantities of Gln, and require high extracellular Gln concentration to grow [2,35]. With both Hs683 and HOG cells, Gln-free incubation, as well as the glutaminolytic enzyme L-asparaginase, suppresses cell growth and leads to cell death, but OD cells exhibit a low EC_50_ for Gln, indicating that even sub-physiological concentrations of the amino acid allow cell survival. In the complete absence of extracellular Gln, the intracellular pool of the amino acid is reduced to less than 1% of control, but, differently from what is reported for other Gln-dependent cells types [1,10,36,37,38,39], 2-OG and pyruvate do not significantly decrease, suggesting that the Gln carbon moiety does not markedly sustain TCA cycle or energy production in OD cells. Consistently, the permeable form of 2-oxoglutarate does not produce any rescue of cell viability during Gln restriction. Although these results exclude the fact that a substantial fraction of Gln is used for maintain the pool of anaplerotic substrates, the quantification of metabolites derived from the amino will require further experiments with ^13^C-labelled Gln.

However, OD cells share with Gln-addicted cancer cells some effects of Gln starvation. For example, Gln depletion lowers mTOR activity although the levels of two known mTORC1 positive regulators (i.e., 2-OG and Leu, [40,41]) are not substantially decreased or, in the case of Leu, even increased (Figure 3a,b). This demonstrates that, at variance with the model proposed by Nicklin et al. [42], in OD cells Gln regulates mTOR independently of Leu content, as also reported in other cell types [6].

Among its different metabolic roles, Gln is of pivotal importance in maintaining the antioxidant pool in several cell models, and it has been shown that Gln addicted cancer cells often have a Gln-dependent redox homeostasis. In these models, glutathione (GSH) partially rescues cells from Gln-starvation-induced cell death [43]. In both HOG and Hs683 cells, Gln-free incubation markedly decreases GSH levels. However, the comparable GSH depletion obtained with BSO, a specific inhibitor of GSH synthesis, does not impair OD cell viability, indicating that GSH shortage is not the main determinant of cell death in Gln-starved OD cells. The marginal role for oxidative stress in the toxicity associated with Gln depletion in OD cells is also consistent with the lack of viability rescue observed upon cystine supplementation of Gln-starved cells (not shown).

Gln-free incubation triggers a clear-cut nutritional stress response in OD cells with the increased phosphorylation of eIF2α, a general reduction of protein synthesis, and the induction of ATF4 targets such as the sodium-dependent Gln transporter SNAT2 and the pro-apoptotic protein CHOP (Figure 4a,b, [44,45]). Transient GS overexpression mitigates the nutritional stress and protein synthesis attenuation, demonstrating that the GS-derived Gln is sufficient to sustain protein synthesis under complete Gln withdrawal.

Interestingly, Gln deprivation also hinders β-catenin expression and activity, known to support OD cell proliferation [27], and decreases the expression of the β-catenin target c-Myc, which is known to upregulate Gln metabolism by increasing glutamine uptake, through ASCT2, and glutaminolysis, through GLS [1,46]. This result excludes that the toxicity observed upon Gln deprivation is mediated by the Wnt/β-catenin pathway activity and, more importantly, suggests that Gln is able to up-regulate its own metabolism through the positive modulation of the pathway.

Given their inability to synthetize Gln, OD cells rely on Gln transporters to take up the amino acid and, indeed, ASCT2, SNAT2 and LAT1 are all expressed in both cell lines. However, the relative expression of ASCT2 and LAT1 is different in Hs683 and HOG cells. Although Hs683 cells express higher levels of ASCT2 mRNA than HOG cells, ASCT2 protein is poorly expressed on the cell membrane and, consistently, Gln influx in these cells is mainly Na^+^-independent. Conversely, ASCT2 protein is mainly on the membrane in HOG cells and most of Gln influx is Na^+^-dependent. As suggested by the Western Blot results, lack of membrane expression of ASCT2 in Hs683 cells is likely attributable to a defective glycosylation of the transporter. The mechanism of this defect is unknown and deserves further investigation. Polet et al. [47] have demonstrated that ASCT2 glycosylation is impaired in glucose-starved cells but ASCT2 expression in Hs683 cells has been studied in the presence of a physiological concentration of glucose (1 g/L). However, similarly to what reported by Polet et al. [47], ASCT2 impairment is also associated with the overexpression of LAT1 in Hs683 cells.

In agreement with previous reports [44,48], Gln deprivation and the consequent nutritional stress lead to an increase of SNAT2 transporter activity. Interestingly, the increase in SNAT2 contribution to Gln uptake, observed in Gln-starved cells, is lowered by GS overexpression, although the basal expression of the transporter is not changed (Figure 7g). This result confirm that GS activity counteracts the nutritional stress caused by Gln starvation.

In various Gln-addicted cancer models, it has been demonstrated that the inhibition of Gln uptake markedly impairs cancer cell growth [9,15,16,36]. In OD cells, however, Gln uptake inhibition, while leads to a marked decrease in Gln content, only modestly reduces cell viability without causing cell death. This observation is consistent with the low EC_50_ for Gln showed by OD cells, which continue to proliferate even if supplied with glutamine concentrations well below the normal physiological levels.

The biological relevance of GS activity has been increasingly recognized in these last few years. In particular, GS role in cancer has been recently reviewed, although straightforward conclusions on the biological significance of GS expression are difficult to reach since the enzyme can be expressed at variable extent in different subtypes of tumors of the same organ or even within a single tumor [35]. Under this regard, OD cells represent a unique human cancer model, which, at difference with other neoplastic cells auxotrophic for Gln [36,49], is Gln dependent but does not rely on Gln anaplerosis for growth and survival.

## 4. Materials and Methods

### 4.1. Cell Culture

The Hs683 and the HOG cell lines, derived from human oligodendrogliomas, were provided by Robert Kiss, University of Brussels, and Glyn Dawson, University of Chicago, respectively [23,34,50]. The U87 glioma cell line was provided by Daniela Parolaro, University of Insubria. Hs683 cells were grown in low-glucose DMEM supplemented with 10% FBS, 4 mM Gln, 25 mM HEPES, and antibiotics (100 U/mL penicillin, and 100 μg/mL streptomycin). HOG and U87 cells were cultured in high-glucose DMEM supplemented with 10 % FBS, 4 mM Gln and antibiotics. After thawing, all cells were used for less than 10 passages.

The mutational status of the *IDH1* and *IDH2* genes was assessed by the diagnostic assay “IDH 1/2^®^ status” (Diatech Pharmacogenetics, Iesi, Italy) based on PCR amplification and pyrosequencing that detects the main somatic mutations of *IDH1* (R132H, R132L, R132C, R132G, R132S) and *IDH2* (R172M, R172T, R172K, R172W, R172G, R172S). No mutation of *IDH1* and *IDH2* was found in either HOG or Hs683 cells. Moreover, the cells did not present the 1p–19q co-deletion, as assessed through Fluorescence In Situ Hybridization (FISH). For FISH, the cell populations were first fixed with 4% paraformaldehyde, incubated with Saline Sodium Citrate buffer (SSC) 2× at 80 °C for 30 min, washed twice with SSC 2× buffer and dehydrated with increasing concentrations of ethanol. Dual-probe hybridization was performed using Orange-labeled locus-specific 1p36 and 19q13 and SpectrumGreen-labeled 1q25 and 19p13 probe sets (Abbott Molecular, Des Plaines, IL, USA), according to the manufacturer’s protocol. Nuclei were counterstained with 4,6-diamidino-2-phenylindole (DAPI) and antifade compound (p-phenylenediamine) and signals assessed under Olympus BX43 fluorescence microscope. For each line, 100 non-overlapping nuclei for orange ‘O’ (marker) and green ‘G’ (reference) signals were assessed. The number of signal patterns for 1p (O) and 1q (G) on one slide, and for 19p (G) and 19q (O) on a different slide, were collected. The signal ratio is considered as the number of orange signals to the number of green signals per cell. A ratio of < 0.8 was considered as a deletion pattern.

### 4.2. RT-PCR Analysis

1 μg of total RNA, isolated with GenElute™ total RNA Miniprep Kit (Sigma, Milan, Italy), was reverse transcribed as described previously [11]. For real time PCR (35 cycles), cDNA was amplified with GoTaq^®^ qPCR Master Mix (Promega, Madison, WI, USA) along with the following primers (5 pmol each): *GLUL* (for 5′-TCATCTTGCATCGTGTGTGTG-3′, rev 5′-CTTCAGACCATTCTCCTCCCG-3′); *RPL-15* (for 5′-GCAGCCATCAGGTAAGCCAAG-3′, rev 5′-AGCGGACCCTCAGAAGAAAGC-3′); *SLC38A2* (for 5′-ATGAAGAAGGCCGAAATGGGA-3′, rev 5′-TGCTTGGTGGGGTAGGAGTAG-3′); *SLC1A5* (for 5′-TGGTCTCCTGGATCATGTGG-3′, rev 5′-TTTGCGGGTGAAGAGGAAGT-3′); *SLC7A5* (for 5′-GTGGACTTCGGGAACTATCACC-3′, rev 5′-GAACAGGGACCCATTGACGG-3′). Quantitative PCR was performed in a 36-well Rotor Gene 3000 (Corbett Research, Rotor-Gene™ 3000, version 5.0.60, Mortlake, Australia). Each cycle consisted of a denaturation step at 95 °C for 30 s, followed by separate annealing (30 s, 55–58 °C) and extension (30 s, 72 °C) steps. Fluorescence was monitored at the end of each extension step. A no-template, no-reverse transcriptase control was included in each experiment. At the end of the amplification cycles a melting curve analysis was added. Data analysis was performed according to the Relative Standard Curve Method. [51] Expression data were normalized to *RPL-15* mRNA expression.

### 4.3. Western Blot Analysis

Cells were lysed in a buffer containing 20 mM Tris-HCl, pH 7.5, 150 mM NaCl, 1 mM EDTA, 1 mM EGTA, 1% Triton, 2.5 mM sodium pyrophosphate, 1 mM β-glycerophosphate, 1 mM Na_3_VO_4_,1 mM NaF, 2 mM imidazole, and a cocktail of protease inhibitors (Complete, Mini, EDTA-free, Roche, Basel, Switzerland). For the enrichment of nuclear proteins, Active Motif Nuclear Extract kit (Active Motif Europe SA, Brussels, Belgium) was used accordingly to the manufacturer’s instructions. Lysates were sonicated for 5 s, and centrifuged at 12,000× *g* for 10 min at 4 °C. After quantification with the Bio-Rad protein assay (Bio-Rad, Hercules, CA, USA), the equivalent of 30 μg of proteins, was mixed with Laemmli buffer 4× (250 mM Tris-HCl, pH 6.8, 8% SDS, 40% glycerol, 0.4 M DTT) and warmed at 95 °C for 5 min. Protein lysates were separated by 10–15% SDS-gel electrophoresis, and transferred onto Immobilon-P membranes (Merck Millipore, Darmstadt, Germany). Non-specific binding sites were blocked with an incubation of 1 h at RT in 5 % non-fat dried milk in TBST solution. The blots were then incubated at 4 °C overnight with the following antibodies diluted in a 5% BSA TBST solution: anti-GS (mouse, monoclonal, 1:1500, BD, Transduction Laboratories, Franklin Lakes, NJ, USA), anti-eIF2α phospho S51 (rabbit, monoclonal, 1:1000, Abcam, Cambridge, UK) anti-p70S6K1 phospho T389 (rabbit, monoclonal, 1:1000, Cell Signaling Technology, Danvers, MA, USA), anti-β-catenin (mouse, monoclonal, 1:1000, DakoCytomation, CA, USA), anti-lamin A/C (mouse, monoclonal, 1:2000, Santa Cruz Biotechnology, Dallas, TX, USA), anti-cyclin D1 (rabbit, monoclonal, 1:1000, Cell Signaling Technology), anti-Myc (rabbit, monoclonal, 1:1000, Cell Signaling Technology), anti-SNAT2 (rabbit, monoclonal, 1:400, Abcam), anti-ASCT2 (rabbit, monoclonal, 1:2000, Cell Signaling Technology), anti-LAT1 (rabbit, monoclonal, 1:1000, Cell Signaling Technology), anti-β-tubulin (mouse, polyclonal, 1:1000, Sigma), anti-GADPH (rabbit, polyclonal, 1:4000, Sigma), anti-β-actin (rabbit, polyclonal, 1:1000, Sigma). After washing, blots were exposed for 1 h at RT to HRP-conjugated anti mouse or anti-rabbit antibody (1:10,000, Cell Signaling Technology). Immunoreactivity was visualized with Immobilon Western Chemiluminescent HRP Substrate (Merck Millipore).

### 4.4. Viability Assay

Cells were seeded in complete growth medium in 24 well-plates and grown for 24 h. Growth medium was then substituted with fresh medium containing the drugs to be tested. For Gln-free incubations, medium was supplemented with dialyzed FBS. Cell viability was assessed with resazurin methods [11], incubating cells in a solution of resazurin (44 μM) solved in serum-free medium. After 2 h, fluorescence was measured at 572 nm with a fluorimeter (EnSpire^®^ Multimode Plate Readers, Perkin Elmer, Boston, MA, USA). Alternatively, cells were counted with a Coulter Z1 particle counter (Beckman Coulter, Brea, CA, USA).

### 4.5. Detection of Apoptosis

Annexin V positive cells were detected with the Muse^®^ cell analyzer (Merck Millipore). Cells were seeded in a 6-well plate. After 24 h growth, medium was substituted with fresh medium containing the drugs to be tested, and incubation prolonged for 24 h. Muse^®^ annexin V and dead cell assay kits were used according to the manufacturer’s instructions. Briefly, treated and control cell suspensions (100 μL) were added to 100 μL of Muse kit reagent 20 min before the measurements, maintaining the mixture in the dark.

Caspase-3 activity was assessed with a Chemicon Caspase-3 Colorimetric Activity Assay Kit accordingly to manufacturer’s instructions. Briefly, cells were centrifuged at 1500 rpm for 10 min; pellets were resuspended in 1× Cell Lysis Buffer and incubated on ice for 10 min. Lysates were then centrifuged for 5 min at 10,000× *g*, and the supernatants were incubated for 2 h at 37 °C with the assay solution. Absorbance at 405 nm was then measured with an EnSpire^®^ Multimode Plate Reader.

### 4.6. Liquid Chromatography Tandem Mass Spectrometry (LC-MS/MS)

Cells were seeded in a 6-well plate. After 24 h growth, medium was substituted with fresh medium in the presence (4 mM) or in the absence of Gln. After experimental times, cells were washed with ice-cold Phosphate Buffered Saline (PBS), and metabolites were extracted with 1 mL cold absolute ethanol. LC analyses were carried out with an Agilent HP 1100 pump coupled with a API4000 triple-quadrupole mass spectrometer (AB SCIEX, Framingham, MA, USA) equipped with a TurboIonSprayTM interface and configured in Selected Reaction Monitoring (SRM) mode adapting a previously published method [52]**.**

### 4.7. GSH Assay

Cells were seeded in complete growth medium in 10 cm^2^ Petri dishes. After 24 h, medium was substituted with fresh medium in the presence (4 mM) or in the absence of Gln. After 19 h, cells were collected, rinsed with PBS, centrifuged at 600× *g* for 5 min, and pellets were lysed. Total GSH content was then measured with a Glutathione assay kit (Sigma) according to manufacturer’s instructions. Data were expressed as GSH/ mg protein.

### 4.8. Protein Synthesis

Cells were treated in the presence (4 mM) or in the absence of Gln, in the presence of Glu (4 mM) and NH_4_Cl (4 mM). L-[4,5-^3^H]Leucine (10 μCi/mL, Amersham Biosciences, UK) was added to the incubation medium during the last 120 min of incubation. At the end of the incubation, cells were washed with ice-cold urea (300 mM) and extracted with 100 μL cold absolute ethanol. Proteins were suspended in 80 μL of 5% sodium-deoxycholate in 1 N NaOH. While 30 μL were used for the determination of total proteins with the Lowry method, scintillation fluid was added to the remaining aliquot, and the incorporated radioactivity counted with a scintillation spectrometer (Wallac Microbeta Trilux counter, Perkin-Elmer, Waltham, MA, USA). Data were expressed as CPM/mg prot/min.

### 4.9. Transient GS Overexpression in Hs683 Cells

Transient overexpression of GS was performed on 30% confluent Hs683 cells using 0.3% Fugene (Promega) in serum-free and antibiotic-free DMEM with 400 ng/mL of either pCMV-GLUL or pCMV scramble vectors (OriGene Products, Rockville, MD, USA).

### 4.10. Glutamine Uptake

The initial influx of Gln was measured in 96-well multidish plates (Falcon, Becton Dickinson Biosciences, Franklin Lakes, NJ, USA) where 15 × 10^3^ cells/well had been seeded 48 h earlier, following the method described in Bianchi et al. [53] Cells were rinsed with 200 μL of Na^+^-free Earle Balanced Salt Solution (EBSS) and incubated at pH 7.4 in EBSS or in a Na^+^-free EBSS, where NaCl was replaced by the chloride salt of *N*-methyl-d-glucamine, supplemented with l-[3,4-^3^H]Gln (5 μCi/mL, Amersham Biosciences) and with either *N*-α-methylaminoisobutyric acid (MeAIB, 20 mM) or threonine (Thr, 5 mM), inhibitors of SNAT2 and ASCT2 transporters, respectively. Although l-glutamyl-*p*-nitroanilide (GPNA) has been repeatedly exploited for ASCT2 inhibition [15,16,36], we did not use this compound given its low specificity [17,54]. After 1 min, cells were washed with ice-cold urea (300 mM), and cell monolayers were extracted with 50 μM of cold absolute ethanol. The extracts were supplemented with 200 μL of scintillation fluid and counted with a scintillation spectrometer. Data were expressed as nmol/mg prot/min.

### 4.11. Immunofluorescence

Cells were seeded on 4-chamber CultureSlides (Falcon, Becton & Dickinson Company, San Jose, CA, USA) at a density of 5 × 10^4^ cells/cm^2^. At the end of the treatments, cells were rinsed twice in PBS and fixed for 10 min in 3.7% paraformaldehyde in PBS. After two further rinses, cells were permeabilized with 0.1% Triton in PBS for 7 min. Cells were then incubated for 1 h in blocking solution (5% of BSA, 10% of goat serum, and 0.3 M glycine in PBS), and then incubated at 4 °C overnight with anti-β-catenin (mouse, monoclonal, DakoCytomation, CA, USA), anti-ASCT2 (rabbit, monoclonal, 1:400, Cell Signaling Technology), or anti-β-catenin (rabbit, polyclonal, 1:250, Abcam) diluted in a 5% BSA PBS solution. After washing, cells were incubated for 1 h with Alexa Fluor 488 anti-mouse IgG and Alexa Fluor 546 anti-rabbit IgG antibodies (Invitrogen, Paisley, UK, 1:400). For nuclear staining, cells were rinsed in PBS and incubated for 30 min with 100 μg/mL RNAase A solution, and then with a 1 μg/mL solution of propidium iodide (PI) in PBS for 10 min. Cells were observed with a confocal microscope Zeiss^®^ 510 LSM Meta (Carl Zeiss SpA, Arese, Milan, Italy), using an oil 60× objective (NA 1.3).

### 4.12. Statistical Analysis

For statistical analysis, two-tail Student’s *t* test for unpaired data or one-way ANOVA were used, as appropriate. GraphPad Prism 5.0™ was used for all the statistical analyses and *p* values < 0.05 were considered statistically significant.

### 4.13. Reagents

Serum was obtained from Lonza, Basel, Switzerland. L-Asparaginase from *E. chrysanthemi* (Erwinase^®^) was a generous gift from Jazz Pharmaceutics. Unless otherwise stated, Sigma (Milan, Italy) was the source of all the other chemicals.
